# The effects of cohousing model on people’s health and wellbeing: a scoping review

**DOI:** 10.1186/s40985-020-00138-1

**Published:** 2020-10-06

**Authors:** Juli Carrere, Alexia Reyes, Laura Oliveras, Anna Fernández, Andrés Peralta, Ana M. Novoa, Katherine Pérez, Carme Borrell

**Affiliations:** 1 Agència de Salut Pública de Barcelona, Pl. Lesseps 1, 08023 Barcelona, Spain; 2 Institut d’Investigació Biomèdica (IIB Sant Pau), C. Sant Quintí 77, 08041 Barcelona, Spain; 3 Department of Experimental and Health Sciences, Universitat Pompeu Fabra, Doctor Aiguader 88, 08003 Barcelona, Spain; 4 CIBER Epidemiología y Salud Pública (CIBERESP), Av. Monforte de Lemos 3-5, Pabellón 11. Planta 0, 28029 Madrid, Spain

**Keywords:** Cohousing, Health, Wellbeing, Quality of life, Psychosocial determinants of health, Scoping review

## Abstract

**Background:**

Housing is a social determinant of health. Extensive research has highlighted its adverse effects on health. However, less is known about the effects of cohousing typology on health, which has the potential to create lively social networks and healthy communities and environments. We report the findings of a scoping study designed to gather and synthesise all known evidence on the relationship between cohousing and wellbeing and health.

**Method:**

Using the scoping review method, we conducted a literature review in PubMed, ProQuest, Scopus, Web of Science, Science Direct and JSTOR in May 2019 and selected articles published from 1960 onwards, with no geographical limit and no design restrictions. Retrieved articles underwent three sequential screening phases. The results were described through a narrative synthesis of the evidence.

**Results:**

Of the 2560 articles identified, we selected 25 full-text articles analysing 77 experiences. All of them were conducted in high-income countries. Ten studies analysed the impact of cohousing on physical and mental health or quality of life and wellbeing. Eight of the 10 studies found a positive association. In addition, 22 studies analysed one or more psychosocial determinants of health (such as social support, sense of community and physical, emotional and economic security) and most found a positive association. Through these determinants, quality of life, wellbeing and health could be improved. However, the quality of the evidence was low.

**Discussion:**

The cohousing model could enhance health and wellbeing mediated by psychosocial determinants of health. However, extreme caution should be exercised in drawing any conclusions due to the dearth of data identified and the designs used in the included studies, with most being cross-sectional or qualitative studies, which precluded causal-based interpretations. Because housing is a major social determinant of health, more evidence is needed on the impact of this model on health through both psychosocial and material pathways.

## Background

Housing is widely recognised as a social determinant of health [1, 2]. Health outcomes are affected by housing affordability, stability, quality and the emotional link to housing, along with the physical and social characteristics of neighbourhoods [3, 4]. While the evidence for the adverse effects of housing on physical and mental health has been reviewed [2, 3, 5, 6], there has been little assessment of the beneficial health effects of housing arrangements where people intentionally live together in a community. Evidence suggests that communal living arrangements reduce feelings of loneliness and increase perceived wellbeing among the senior population compared with residents living in single arrangements [7, 8].

Among communal living arrangements, here we review the cohousing model. The existing literature on cohousing is characterised by a certain degree of ambiguity and overlap between different terms and experiences. However, there is consensus among different authors in defining cohousing as a form of community living that contains a mix of private and communal spaces with substantial self-managed common facilities and activities aimed at everyday living [9–11].

To date, the evidence suggests that cohousing decreases isolation in seniors, positively impacts inhabitants’ quality of life and benefits physical and mental health [12, 13]. Among intergenerational housing residents, cohousing also increased mutual support and created a sense of community among residents [14–16]. These feelings could be extended to the neighbourhood by increasing the sense of community beyond the boundaries of cohousing, resulting in improved wellbeing among residents [17]. However, there are fewer studies on the physical and mental health effects in intergenerational populations, and the results appear unclear.

The cohousing model was created in Denmark in the early 1970s as an innovative form of collective housing and later spread to other northern European countries, the USA [18] and other latitudes such as Uruguay [19]. In recent years, cohousing has re-emerged in the USA, Europe, Australia, New Zealand and Japan [16, 20, 21]. This re-emergence has been associated with a growing desire for a sense of belonging, to experience more connection with the community and an increasing rejection of dominant consumption patterns [22]. In addition, it has been boosted by the lack of affordable housing and poor rental conditions and has been presented as a potential alternative to conventional tenure arrangements [16, 18, 23]. Research on the cohousing model has so far covered different topics. For example, the architectural features and physical layout of buildings [15, 24, 25], the environmental sustainability practices in communities [26–29] and self-management and decision processes [30]. Few studies, however, have explored the different tenure modalities [23, 31], their ability to promote social capital [16] and whether social housing could be an opportunity for municipalities to promote socially inclusive urban development [32].

The cohousing model has also attracted the attention of public health [33]. The driving motivation is to provide evidence of the increased quality of life among people living in cohousing, which is often an objective of cohousing projects but is rarely assessed, to facilitate evidence-based decision-making. From the point of view of health promotion, the expansion of this model is related to the need to respond to the phenomenon of social isolation through community-based housing models that promote healthy built environments and foster people’s social cohesion [33]. Furthermore, the cohousing model is credited with the ability to improve the affordability of housing [18], which is known to be beneficial to the wellbeing of the population.

Although there is only a modest number of studies on cohousing and health, wellbeing, or quality of life, research in this field is slowly increasing. The present review aims to gather and synthesise all the known evidence on the relationship between cohousing and health and wellbeing.

## Methods

We conducted a literature review using the scoping review method. This method has an exploratory character and is indicated to synthesise the scientific knowledge and identify the key concepts and research gaps in areas of study with little available scientific evidence [34]. The literature search was performed in May 2019, and we consulted databases in social sciences, architecture and health: PubMed, ProQuest, Scopus, Web of Science, Science Direct and JSTOR. Full details of the search strings used for the various databases are shown in Supplementary 1. Note that to capture articles related to cohousing and health, the search syntax was adapted to each database.

The inclusion criteria were peer-reviewed documents: (1) studies of cohousing living arrangements where communal spaces and/or common facilities or services are available and self-managed; (2) documents published after 1960, as the cohousing model started in Denmark in the 1960s; (3) documents that analysed at least one health outcome such as physical or mental health, self-perceived health, or wellbeing, or assessed psychosocial determinants of health, such as social support, social isolation, life satisfaction, happiness, or sense of community; and (4) documents written in English, Spanish, French, German or Italian. We excluded books and conference communications and studies without an available summary.

We applied three sequential phases of document screening to the list of documents retrieved by the searches. In phases 1 and 2, we screened the documents by title/abstract and full text, respectively. In phase 3, we manually retrieved additional documents that also met the inclusion criteria from the reference lists of the documents selected, as well as the literature identified after expert consultation and repeated the same screening procedure. Details of the experts contacted are shown in Supplementary 2. To ensure internal validity, we triangulated the results as follows: phases 1 and 2 were carried out separately by three independent pairs of researchers, who discussed the inclusion/exclusion of the documents that generated doubts. A third researcher was included if there were still doubts. For the articles retrieved in phase 3, we replicated the same selection and triangulation process, but only one pair of researchers participated.

For each selected document, we extracted the following information: (a) characteristics of the studies, (b) characteristics of cohousing projects studied and (c) health-related outcomes. The included characteristics of the studies were year of publication, type of methods and number of projects studied. The characteristics of cohousing projects were year of cohousing project creation, country of cohousing project, age target and co-ownership tenure. The health-related outcomes analysed were grouped in *(i)* self-perceived physical and mental health, *(ii)* quality of life and wellbeing and *(iii)* psychosocial determinants of health, which include social support, social isolation, sense of community and sense of security and safety.

Finally, we provide an in-depth description of the main health-related findings of cohousing projects studied.

## Results

**Fig. 1:**
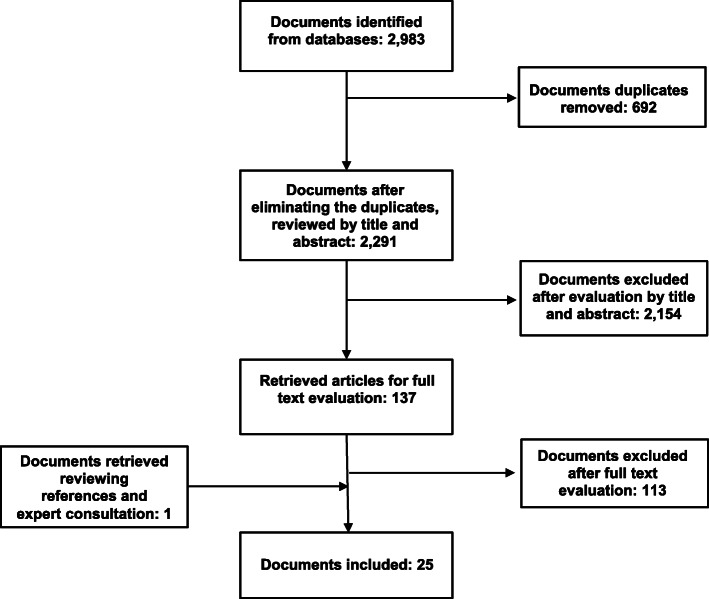
Flowchart of the search and selection procedure of documents

The search and selection process and the documents included are summarised in Fig. 1. The search resulted in a total of 2983 documents: 516 in PubMed, 31 in ProQuest, 913 in Scopus, 922 in the Web of Science, 93 in Science Direct and 508 in JSTOR. Of these, 269 were duplicates. In all, 2291 documents were excluded after reviewing the title and abstract. After reading the full text of the remaining 137 documents, 24 were included. We reviewed the references from these documents, and we contacted experts by e-mail. Eight experts offered information or referred us to other experts or specialised organisations. As a result, 41 new documents were retrieved, and 1 was selected for the review. A total of 25 documents were finally included in the scoping review.

Table 1 shows the main characteristics of the documents selected. The publication rate tended to increase over time, with 24% of the documents published between 2001 and 2010, and 64% of documents published after 2010. Regarding the study designs, 20% were quantitative studies, all were cross-sectional and two of them had a comparison group. A total of 40% were qualitative studies, and the most commonly used techniques were in-depth interviews (*n* = 6) and semi-structured interviews (*n* = 4), while four studies applied more than one technique. Mixed methods were employed by 40% of the studies. Three of them used longitudinal designs. Two studies used a comparison group in the quantitative approach, while in-depth interview was the most commonly applied technique in the qualitative approach.

**Table 1:** Description of studies included by year of publication, country, methods and number of cohousing projects studied, as well as description of cohousing projects by year of creation, country, age target and co-ownership status dimension and the health outcomes analysed

	*N*	(%)
**a) Characteristics of the studies**	25	100.0
**Year of publication**		
Before 1990	0	0.0
1990-2000	3	12.0
2001-2010	6	24.0
After 2010	16	64.0
**Methods**		
Quantitative	5	20.0
- Cross-sectional with comparison group	2	
- Cross-sectional without comparison group	3	
Qualitative*	10	40.0
- In-depth interviews	6	
- Semi-structured interviews	4	
- Group discussion	1	
- Participant observation	3	
Mixed methods	10	40.0
- Quantitative		
- Longitudinal without comparison group	3	
- Cross-sectional with comparison group	2	
- Cross-sectional without comparison group	5	
- Qualitative**		
- Longitudinal in-depth interviews	3	
- In-depth interviews	4	
- Semi-structured interviews	3	
- Group discussion	1	
- Participant observation	1	
**Number of projects included per document**		
1	9	36.0
2-5	11	44.0
+5	3	12.0
Unknown	2	8.0
**b) Characteristics of cohousing projects studied**	77	100.0
**Year of cohousing projects creation**		
Before 1990 1990-2000	19 17	25.0 22.0
2001-2010	30	39.0
After 2010	4	5.0
Unknown	7*	9.0
**Country of cohousing projects**		
Canada	18	23.0
Europe	41	53.0
USA	18	23.0
**Age target**		
Intergenerational	48	62.0
Elderly	29	38.0
**Co-ownership tenure**		
No	50	65.0
Yes	27	35.0
c) Health related outcomes***	25	100.00
Self-perceived physical and mental health	4	16.0
Quality of life and well-being	6	24.0
Psychosocial determinants of health		
-Social support	22	88.0
- Social isolation	5	20.0
-Sense of community	11	44.0
**d) Effects of cohousing on the health outcomes analysed**	25	
Self-perceived physical and mental health		
- Beneficial	3	
- Neutral	1	
- Detrimental	1	
Total****	4	
Quality of life and well-being		
- Beneficial	5	
- Neutral	1	
- Detrimental	0	
Total	6	
Psychosocial determinants of health		
- Beneficial	22	
- Neutral	3	
- Detrimental	2	
Total****	22	

*Four studies used more than one qualitative research technique

**Two studies used more than one qualitative research technique

***Several studies considered more than one type of health-related outcomes; the percentage corresponds to the number of studies in which the health outcome was analysed

****Several studies reported more than one type of health-related outcomes; furthermore, three studies reported different health effects on the same health outcome because they analysed different subpopulations or different indicators

The proportion of articles examining between two and five cohousing living arrangements projects per document was high (44%). The 25 studies examined a total of 77 projects. A quarter (25%) of the cohousing projects studied were created before 1990, 22% between 1990 and 2000, 39% between 2001 and 2010 and only 5% after 2010. Most projects (53%) were conducted in European countries, with the vast majority being conducted in northern countries, followed by the USA (23%) and Canada (23%). Cohousing projects target predominantly the intergenerational population (62%), and tenure was co-ownership (35%).

Regarding health outcomes, four studies analysed physical and mental health through self-perceived assessment, and one of them measured healthcare requirements. Six studies analysed quality of life and wellbeing. Twenty-two selected studies included the psychosocial determinants of health. Finally, beneficial effects of cohousing on health outcomes were reported in three studies analysing physical and mental health, in five analysing quality of life and wellbeing and in twenty assessing psychosocial determinants of health.

### Cohousing-related health effects

Table 2 describes the health outcomes and the psychosocial determinants of health of the cohousing projects studied, and Table 3 summarises their observed effects.

**Table 2:** Description of the main health-related results found in the cohousing projects

1st Author, year	Aim	Characteristics of the projects	Methodology (design or technique)	Instruments	Main results
Altus and Mathews, 2002	To compare the satisfaction of rural senior housing cooperative and rental apartments members.	**Cohousing country:** USA **Age target:** Elderly **Co-ownership:** Yes	**Quantitative** (cross-sectional with comparison group) **N**: 39 cooperative residents; 48 rental apartments **Number of projects studied:** 3	Quality of life measured by well-being index with 12 items related to safety, happiness, life satisfaction, friends, physical health, and psychological health**.**	**QUALITY OF LIFE AND WELL-BEING** There are no significant differences between cohousing cooperative residents and rental apartment residents.
Bamford, 2005	To explore the physical structures and experiences of older people living in cohousing.	**Cohousing country:** Denmark and Netherlands **Age target:** Elderly **Co-ownership:** Yes	**Qualitative** (cross-sectional; in-depth interviews) **N**: unknown **Number of projects studied:** 2	Interview guide not provided.	**PSYCHOSOCIAL DETERMINANTS OF HEALTH** **Social support:** Physical structures facilitate social relations and benefit the sense of security. Different people go on outings together and in general “appreciate the social contact” and the possibility of “help or assistance” in time of need, but they remain keen to preserve their autonomy.
Choi and Paulsson, 2011	To evaluate the social support and quality of life in Swedish cohousing units.	**Cohousing country:** Sweden **Age target:** Intergenerational **Co-ownership:** No	**Quantitative** (cross-sectional without comparison group) **N:**241 **Number of projects studied:** 12	Life satisfaction with housing was measured by 6 items with Likert-scale of 3 points and 5 points.	**QUALITY OF LIFE AND WELL-BEING** Increase life satisfaction among people living in cohousing compared to their own experience before and after moving. Most respondents indicated high level of satisfaction and happiness with their lives in cohousing. People over 60 thought they lived better than others of their age living in conventional housing. **PSYCHOSOCIAL DETERMINANTS OF HEALTH** **Social support:** mutual support in cohousing communities is perceived greater than in conventional ones.
Cooper and Rodman, 1994	To assess how differences in physical design and the ability of residents to control the environment affect their quality of life.	**Cohousing country:** Canada **Age target:** Intergenerational **Co-ownership:** Yes	**Mixed methods** Quantitative (cross-sectional without comparison group) Qualitative (cross-sectional; in-depth interviews) **N**:62 with disabilities; 241without disabilities **Number of projects studied:** 16	**Quantitative:** Questionnaire conducted to gather data on levels of satisfaction, quality of life, participation, control, and social integration. **Qualitative:** interview guide not provided.	**QUALITY OF LIFE AND WELL-BEING** The social control perceived by residents over their residential environment was more important than their perceived physical control (accessibility) in explaining the perceived quality of life.
Fromm, 2000	To determine whether residents had achieved their stated goal of "creating a sense of community" through cohousing; and, if achieved, their satisfaction with it.	**Cohousing country:** USA **Age target:** Intergenerational **Co-ownership:** No	**Mixed methods** Quantitative (cross-sectional without comparison group Qualitative (cross-sectional; semi-structured interviews) **N**:85 units **Number of projects studied:** 3	**Quantitative:** information on the questions not provided **Qualitative:** interview guide not provided.	**PSYCHOSOCIAL DETERMINANTS OF HEALTH** **Social support:** 100% of cohousing residents would feel comfortable asking neighbours to help with tasks or errands if they were ill, in their previous conventional housing only 40% reported the same possibility. **Sense of community:** The residents have a much stronger sense of community within cohousing than in their previous neighbourhood**.** **Sense of security:** There are feelings of security within the cohousing community.
Glass, 2009	To describe a resident-managed elder-only cohousing community focusing on mutual support and affordable housing.	**Cohousing country:** USA **Age target:** Elderly **Co-ownership:** No	**Mixed method** Quantitative (longitudinal without comparison group) Qualitative (longitudinal; in-depth interviews) **N:**33 **Number of projects studied:** 1	**Quantitative:** Physical and mental health measured by self-perceived physical health and self-perceived mental health. **Qualitative**: interview guide not provided.	**SELF-PERCEIVED PHYSICAL AND MENTAL HEALTH** 19% of project participants report improvements and 13% reported worsening in physical health compared to one year ago. 28% reported improvements in mental health and 3% reported worsening mental health. **PSYCHOSOCIAL DETERMINANTS OF HEALTH** **Social support:** Residents count on their neighbours for help with household and personal care. **Sense of community:** The sense of community and mutual support are perceived as important reasons for living in a cohousing.
Glass, 2012	To describe the health status of three elder-cohousing projects.	**Cohousing country:** USA **Age target:** Elderly **Co-ownership:** No	**Mixed methods** Quantitative (longitudinal without comparison group) Qualitative (longitudinal; in-depth interviews) **N:**58 **Number of projects studied:** 3	**Quantitative:** General health was measured by self-perceived health. Information on mental health scale used not provided. **Qualitative:** interview guide not provided.	**SELF-PERCEIVED PHYSICAL AND MENTAL HEALTH** The majority had good physical and mental health and it remained the same a year later living in the cohousing
Glass, 2013	To evaluate how cohousing projects, influence the ageing of older people.	**Cohousing country:** USA **Age target:** Elderly **Co-ownership:** No	**Mixed methods** Quantitative (longitudinal without comparison group) Qualitative (longitudinal without comparison group with interviews and participant observation) **N**:43 **Number of projects studied:** 1	**Quantitative:** information on the questions not provided. **Qualitative:** interview guide provided. Core questions related to the review: Does living in this community affect how you think and feel about the aging process and any challenges that can come with that process? If so, how? How is the mutual support working out? Have your expectations changed?	**PSYCHOSOCIAL DETERMINANTS OF HEALTH** **Social support:** Residents were willing to help others, ask for help when needed and accept help. **Sense of community:** Development and satisfaction of a sense of community and mutually supportive processes. Bring prospective residents together regularly while the buildings are under construction to begin to build a sense of community and to discuss expectations about the community.
Glass, 2016	To determine if neighbourhoods, each based on the cohousing model promote development of social resources for their residents.	**Cohousing country:** USA **Age target:** Elderly **Co-ownership:** No	**Quantitative** (cross-sectional without comparison group) **N**:59 **Number of projects studied:** 3	Social networks measured by Lubben Social Network Scale. Neighbouring support through four items. Satisfaction with the neighbourhood community measured by seven items.	**PSYCHOSOCIAL DETERMINANTS OF HEALTH** **Social support:** The mutual support most frequently reported was sharing of knowledge to help someone (informational), lending/borrowing things (functional), and listening/supporting when someone had a personal problem (emotional). **Social isolation:** Living in an intentional neighbourhood reduces social isolation by increasing social resources. **Sense of community:** Participants were very dissatisfied with sense of community or feelings part of community. **Sense of security:** 77,6% were very satisfied with security and safety from crime.
Glass and Vander Plaats, 2013	To assess life in co-housing and the beneficial results of living together in relation to ageing.	**Cohousing country:** USA **Age target:** Elderly **Co-ownership:** No	**Mixed methods** Quantitative (cross-sectional without group) in 2012 Qualitative (cross-sectional; in-depth interviews) in 2009 **N**:31 **Number of projects studied:** 1	**Quantitative:** information on the questions not provided. **Qualitative:** interview guide provided. Core questions related to the review: Why did you choose to move here? Why did you choose an elder-only community? How is the mutual support working out? Have your expectations changed?	**PSYCHOSOCIAL DETERMINANTS OF HEALTH** **Social support:** An increase of mutual support, sense of safety and acceptance of aging was perceived. Residents explained that they were prepared to help each other and learning to age well together. The 89% say there is more mutual support in cohousing. **Social isolation** Over 90% of residents reported agreement or satisfaction on feeling safe, less worry and lessening of social isolation. **Sense of security** Residents showed feelings of security related to mutual support, socialising and companionship among residents. All residents indicated that living in cohousing makes feeling safe.
Jolanki and Vilkko, 2015	To study what a “sense of community” meant to the residents and how a sense of community becomes visible in daily life.	**Cohousing country:** Finland **Age target:** Elderly **Co-ownership:** No	**Mixed methods** Quantitative (cross-sectional without comparison group) Qualitative (cross-sectional; semi-structured interviews and groups discussions) **N**:6 discussants and 41surveyed **Number of projects studied:** 1	**Quantitative:** information on the questions not provided. **Qualitative:** Core questions related to the review: What kind of meanings are given to a “sense of community” by the residents of the co- housing community? How does a “sense of community” become visible in the daily life of the community, according to the residents?	**PSYCHOSOCIAL DETERMINANTS OF HEALTH** **Social support:** Community as a source of social and practical support for residents. **Sense of community:** Sense of community understood as a sense of togetherness, belonging and trust, created through community activities, doing things together, and have mutual support. **Sense of security:** Trust in the community as a collective unit that supported its residents, trust in other residents, and always having someone to turn to create a sense of security and safety.
Kehl and Then, 2013	To assess the effects of multi- generation cohousing developments on the residents, health conditions and social support.	**Cohousing country:** Germany **Age target:** Intergenerational **Co-ownership:** No	**Quantitative** (Cross-sectional with comparison group) N: 313 program group; 428 control group **Number of projects studied:** 4	General health was measured by self-perceived health Care level was measured with scale from 0 points to 3 points. Social support measured with scale from 0 points to 5 points.	**SELF-PERCEIVED PHYSICAL AND MENTAL HEALTH** No significant differences in subjective health assessment. 13% of the programme group respondents are in need of care compared to 22% in the control group. **PSYCHOSOCIAL DETERMINANTS OF HEALTH** **Social support:** Cohousing participants show more social support and social cohesion than the control group.
Labit, 2015	To explore the effects of cohousing on the quality of life of elderly people by focusing on personal autonomy and community solidarity.	**Cohousing country:** 2 Germany, 1 Sweden, 2 UK. **Age target:** Intergenerational **Co-ownership:** No	**Qualitative** (cross-sectional; semi-structured interviews, participants observation and photographic record) **N**: 30 with residents aged over 50 **Number of projects studied:** 5	Interview guide not provided.	**QUALITY OF LIFE AND WELL-BEING** Cohousing is considered a good housing option for older people as it improves the quality of their lives by focusing on personal autonomy and community solidarity. **PSYCHOSOCIAL DETERMINANTS OF HEALTH** **Social support:** Mutual assistance is often described as the result of affinity, although mutual assistance and solidarity between generations was also reported. **Sense of security:** Solidarity and good neighbourly relations foster a sense of security, something that was most evident in senior participants.
Labit and Dubost, 2016	To learn about the experiences of residents living in a model of cohousing based on solidarity between elderly people and families in Germany.	**Cohousing country:** Germany **Age target:** Intergenerational **Co-ownership:** No	**Qualitative** (cross-sectional; semi-structured interviews) **N:** 10 Cologne; 8 Berlin **Number of projects studied:** 2	Interview guide not provided.	**PSYCHOSOCIAL DETERMINANTS OF HEALTH** **Social support:** Creation of a strong attachment in mothers (single or not) because the community makes daily life easier Intergenerational solidarity was not evident although seniors reported receiving help when they requested. Older participants are discouraged by conflicts (budget management and maintenance of common areas)
Markle et al., 2015	To explore cohousing residents’ experience of social support in the USA.	**Cohousing country:** USA **Age target:** Intergenerational **Co-ownership:** Yes	**Mixed method** Quantitative (cross-sectional with comparison group) Qualitative (cross-sectional; semi-structured interview) **N:** 60 living in cohousing; 65 not living in cohousing **N:** 10 interviews **Number of projects studied:** Unknown	**Quantitative:** Social support was measured with three scales: Social Provisions Scale (SPS), Social support given (SSG) and Social support received (SSR). **Qualitative:** interview guide provided. Core questions related to the review: How do you give and/or receive support from other people in your cohousing community? How has living in cohousing impacted or changed your life?	**PSYCHOSOCIAL DETERMINANTS OF HEALTH** **Social support:** People who live in cohousing receive and give more social support than people who do not. Moreover, cohousing participants indicated that they felt more supported in cohousing compared to previous living situations. **Sense of community:** Elderly are aware that they can receive help from the community in their ageing and with their health problems. The sense of community was a reason for joining cohousing.
Motevasel, 2006	To know the expectations and differences between residents in rental apartments and tenant-owned housing cooperative.	**Cohousing country:** Sweden **Age target:** Elderly **Co-ownership:** Yes (2/4)	**Qualitative** (cross-sectional; in-depth interviews with comparison group) **N**: 16 seniors in rental apartments; 12 seniors in cooperatives **Number of projects studied:** 4	Interview guide provided. Core questions related to the review: Why have the residents chosen senior housing? What are the differences between residents in rental apartments and tenant-owned housing cooperatives? What advantages and disadvantages do the residents think that senior housing has?	**PSYCHOSOCIAL DETERMINANTS OF HEALTH** **Social support:** Socialisation, activities and community were perceived positive both, in the rental apartments and the cohousing cooperatives. **Social isolation**: The architectural design forced socialisation and it was not always a positive experience. Nevertheless, it avoided the social isolation of the elderly who were sick and frail. **Sense of community:** The informants did not think that they have chosen their present housing out of a desire for security or social community. However, they have come to appreciate that there is a community in the senior housing**.**
Nusbaum, 2010	To understand how creating and living in an elder co-housing community has impacted residents' sense of autonomy, a critical component of psychological well-being in old age.	**Cohousing country:** USA **Age target:** Elderly **Co-ownership:** No	**Qualitative** (cross-sectional; in-depth interviews) N:10 **Number of projects studied:** 1	Interview guide provided. Core questions related to the review: Can you tell me about a typical day here at Capitol Court? How is living at Capitol Court going for you? How important is autonomy to you, or the meaning of deciding what you want in your life? Have you always been that way, or is that a quality that has become more important as you have gotten older?	**QUALITY OF LIFE AND WELL-BEING** The participants deeply valued both their own autonomy and their fellow residents. Participants reported their autonomy was not compromised by functioning as a cohesive group, indeed various aspects of group life foster individual autonomy. Living in an environment with a high degree of autonomy favours opportunities to participate and contribute, resulting in an improvement in different competences and skills. **PSYCHOSOCIAL DETERMINANTS OF HEALTH** **Social support:** Participants cared greatly about the quality of their relationships with each other, and yet they also cared about quantity of time spent together.
Pedersen, 2015	To describe how the residents have adapted to the individual and collective challenges in a Danish senior cohousing	**Cohousing country:** Denmark **Age target:** Elderly **Co-ownership:** No	**Mixed method** Quantitative (cross-sectional without group comparison) Qualitative (cross-sectional; in-depth interviews) **N:** 643 surveyed and unknown interviews) **Number of projects studied:** Unknown	**Quantitative:** The questionnaire collected topics about the social interaction in the facilities and residents’ perceptions of the advantages and challenges of living in a senior co-housing community. Measures not provided. **Qualitative:** Core questions related to the review: Why did the residents choose to spend their old age in a co-housing community? Was it difficult to recruit new members for the board of residents?	**PSYCHOSOCIAL DETERMINANTS OF HEALTH** **Social support:** Social networks and satisfaction with housing increased compared to the previous housing situation. Interviews indicated that social and practical activities played an important role in residents' lives and promoted closer friendships. **Sense of security:** Most residents valued the sense of security they gained from living in an environment where people knew each other well.
Philippsen, 2014	To know the degree of social integration of the residents into their residential group and the mechanisms by which the integration takes place.	**Cohousing country:** Germany **Age target:** 6 Intergenerational and 1 Senior **Co-ownership:** Yes (1/7 projects)	**Quantitative** (cross-sectional without comparison group) **N:** 220 adults **Number of projects studied:** 7	The questionnaire comprises three thematic blocks with 71 questions: 1) questions about life in the housing project, 2) questions about life situation and personal relationships and 3) general questions about the person. Social support was assessed by the study of social networks and 10 questions based on the Fischer instrument and adapted to the special conditions in housing projects, covering instrumental and emotional support.	**PSYCHOSOCIAL DETERMINANTS OF HEALTH** **Social support:** 83-95% of residents have obtained instrumental or emotional support and 75-88% have given support. 93% of the inhabitants believe that it is possible to make close friendships in cohousing. Although, elderly residents have more friendships than younger ones. Residents who regularly attend cohousing social gatherings are much more likely to be friends than residents who attend meetings only occasionally or never, the closer residents felt, the more likely they were to support each other, both emotionally and instrumentally. Residents of all seven projects indicated more instrumental social support (help with cures, housework, etc.) than emotional support.
Rodríguez-Alonso and Argemir, 2017	To explore personal autonomy, organisation of physical space and collective self-management in the senior cohousing cooperative.	**Cohousing country:** Spain **Age target:** Elderly **Co-ownership:** No	**Qualitative** (cross-sectional; in-depth interviews, participant observation and a group discussion workshop) **N:** 29 **Number of projects studied:** 1	Interview guide not provided. Group discussion workshop focus on reasons for dismissing family and senior residence as primary options for care and how care is materialised in the cohousing.	**QUALITY OF LIFE AND WELL-BEING** The ability to decide and manage the forms and timing of self-care promotes physical, emotional, and social well-being. **PSYCHOSOCIAL DETERMINANTS OF HEALTH** **Social support:** Autonomy is only possible through collective support and social relations of solidarity. Physical spaces also allow for social interaction that leads to emotional bonds.
Ruiu, 2015	To assess whether cohousing communities (the case of Threshold Centre) might generate positive effects in terms of social housing.	**Cohousing country:** USA **Age target:** Intergenerational **Co-ownership:** Si	**Qualitative** (cross-sectional; semi-structured interviews and cognitive maps) **N:**18 **Number of projects studied:** 1	Interview guide not provided instead a list of topics was provide, topics related to the review were: Decision-making process; Physical layout and social life; Social dynamics and privacy (public and private spaces); Shared values and “ideologies” Relations with the outside; Safety.	**PSYCHOSOCIAL DETERMINANTS OF HEALTH** **Social support:** There is informal mutual support among residents and in the neighbourhood **Sense of community:** Participation in all stages of the process, in addition to self-management, contributes to a sense of community Designing and managing common spaces helps to define community life and a collective perception **Sense of security:** High sense of security thanks to the physical layout of the community.
Tchoukaleyska, 2011	To know how cohousing communities can reduce the risks associated with living in an urban context and are a desirable place to raise children.	**Cohousing country:** Canada **Age target:** intergenerational **Co-ownership:** Yes	**Qualitative** (cross-sectional; semi-structured interviews) **N**:5 residents (three family interviews) **Number of projects studied:** 1	Interview guide not provided	**PSYCHOSOCIAL DETERMINANTS OF HEALTH** **Social support:** Cohousing encourage social relationship, social support among families and allows children to meet friends of different ages. Many participants also reported the exchange of practical, pragmatic support within their community, involving sharing objects, borrowing cars, or offering rides, and caring for each other’s homes, plants, and pets while they were away **Sense of community:** There is a sense of community and is a reason behind the selection of cohousing for their families, parents indicate. **Sense of security:** There were a common desire to provide a family-oriented environment and emotionally and physically secure for children.
Tyvimaa, 2011	To discuss residents’ views of social and physical environments in a cohousing and in a senior housing setting in Finland.	**Cohousing country:** Finland **Age target:** Elderly **Co-ownership:** No	**Mixed methods** Quantitative (cross-sectional with comparison group) Qualitative (cross-sectional; in-depth interviews) **N**: 34 group intervention; 64 group comparison **N**:14 interviews **Number of projects studied:** 2	**Quantitative:** information on the questions not provided. **Qualitative:** interview guide not provided.	**PSYCHOSOCIAL DETERMINANTS OF HEALTH** **Social support:** Well-designed common areas activate residents to socialise and organise activities. They use their common areas more actively than residents in conventional housing. 29% of cohousing residents and 14.9% of senior housing residents said they met with neighbours at least once. Social networking is an integral component of happiness within the housing setting. **Social isolation:** The residents had experienced loneliness or social isolation before moving into co-housing, after moving that sense disappears. **Sense of community:** The sense of community was a reason for choosing to live in cohousing. Activities organised together connect the residents together and increase the feeling of sense of community
Wasylishyn and Johnson, 1998	To develop an understanding of the experiences of women living in a new housing co-operative built exclusively for unattached, low income women of middle age.	**Cohousing country:** Canada **Age target:** Intergenerational **Co-ownership:** Yes	**Qualitative** (cross-sectional; in-depth interview; participants observation) **N:**10 women **Number of projects studied:** 1	Interview guide not included.	**SELF-PERCEIVED PHYSICAL AND MENTAL HEALTH** A deterioration in physical and/or mental health was perceived after moving into the co-operative. **PSYCHOSOCIAL DETERMINANTS OF HEALTH** **Social support:** The increased sense of control and social support inherent in community life were not immediately apparent. **Sense of community:** Women perceived themselves as a diverse group without a common identity. **Sense of security:** Less financial stress and more sense of safety.
Williams, 2005	To know how the physical design and management of a cohousing influences the social interaction of the residents.	**Cohousing country:** USA **Age target:** Intergenerational **Co-ownership:** No	**Qualitative** (cross-sectional; activity diaries, in-depth interviews, and participants observation) **N:**98 **Number of projects studied:** 2	Interview guide not provided	**PSYCHOSOCIAL DETERMINANTS OF HEALTH** **Social support:** The number and diversity of social activities organised in a community seem to affect levels of social interaction. The management of indoor communal facilities was also shown to influence usage and social interaction. Meetings could potentially provide the opportunity for more social interaction amongst residents. Density (proximity) and layout, the division of public and private space and the quality, type and functionality of communal spaces appear to be the key design factors influencing social interaction in cohousing developments

**Table 3:** Number of studies showing a beneficial, neutral or detrimental effect of cohousing on the health outcomes analysed

	Beneficial	Neutral	Detrimental
**Self-perceived physical and mental health**^**a**^	3 (Glass, 2012, 2009; Kehl and Then, 2013)	1 (Kehl and Then, 2013)	1 (Wasylishyn and Johnson, 1998)
**Quality of life and well-being**	5 (Choi and Paulsson, 2011; Cooper and Rodman, 1994; Labit, 2015; Nusbaum, 2010; Rodríguez-Alonso and Argemir, 2017)	1 (Altus and Mathews, 2002)	
**Psychosocial determinants of health**			
**Social support**^**b**^	20 (Bamford, 2005; Choi and Paulsson, 2011; Fromm, 2000; Glass, 2016, 2013, 2009; Glass and Vander Plaats, 2013; Jolanki and Vilkko, 2015; Kehl and Then, 2013; Labit, 2015; Labit and Dubost, 2016; Markle et al., 2015; Nusbaum, 2010; Pedersen, 2015; Philippsen, 2014; Rodríguez-Alonso and Argemir, 2017; Ruiu, 2015; Tchoukaleyska, 2011; Tyvimaa, 2011; Williams, 2005)	3 (Labit and Dubost, 2016; Motevasel, 2006; Wasylishyn and Johnson, 1998)	
**Social isolation**	5 (Glass, 2016; Glass and Vander Plaats, 2013; Motevasel, 2006; Tyvimaa, 2011; Wasylishyn and Johnson, 1998)		
**Sense of community**^**c**^	10 (Fromm, 2000; Glass, 2016, 2013, 2009; Jolanki and Vilkko, 2015; Markle et al., 2015; Motevasel, 2006; Ruiu, 2015; Tchoukaleyska, 2011; Tyvimaa, 2011)		2 (Markle et al., 2015; Wasylishyn and Johnson, 1998)
**Sense of security**	9 (Bamford, 2005; Fromm, 2000; Glass, 2016; Glass and Vander Plaats, 2013; Jolanki and Vilkko, 2015; Pedersen, 2015; Ruiu, 2015; Tchoukaleyska, 2011; Wasylishyn and Johnson, 1998)		

^a^Kehl and Then, 2013. Article is included in the beneficial and neutral effects boxes. The article reported less use of health care service but no difference on self-perceived health differences

^b^Labit and Dubost, 2016. Article is included in the beneficial and neutral effects boxes. The article reported more social support among inhabitants of the same generation and less evident social support across generations

^c^Markle et al., 2015. Article is included in the beneficial and detrimental effects boxes. The article reported contradictory effects that cannot be classified as neutral

### Self-perceived physical and mental health

Four studies aimed to assess the impact of cohousing on self-perceived physical and mental health. Two of them used mixed methods [35, 36], one used quantitative method [37] and the other applied a qualitative design [38]. The former did not provide the guide used in the interviews. The three studies employing survey-based quantitative approaches used a validated question—self-perceived health—and no other scales. Mental health was assessed one-dimensionally, with no scales measuring more than one mental health domain.

Self-perceived physical and mental health in two senior projects increased in the follow-up [35, 36], while in the intergenerational projects the effect on health was less clear. No significant differences were observed in self-perceived physical health in the single article that used a comparison group, although cohousing residents reported less need for health and social care services [37]. In contrast, a project for low-income middle-aged women found that residents reported a deterioration in their physical and mental health after they moved to the cohousing cooperative [38].

### Quality of life and wellbeing

All but one study assessing quality of life and wellbeing reported positive benefits for cohousing residents, two using qualitative methods [39–41], one mixed method [42] and another one quantitative method [43]. The single study that did not report significant benefits used a quantitative method with a comparison group [44]. The latter compared cohousing residents who were owners with residents in congregated apartments who were tenants. Only one study using a qualitative approach provided information on the interview guide used [39]. Two of the studies employing quantitative approaches provided information on the questions used to assess quality of life [43, 44]; none of the studies used the same scale.

The studies that found improvements in quality of life and wellbeing explained these gains by increased autonomy, increased opportunities to participate in the community and greater solidarity among cohousing residents, in both senior [39–41] and intergenerational [42, 43] projects. One of these intergenerational projects was based on Canadian non-profit cohousing cooperatives for people with functional diversity [42]. In this case, improvements in the quality of life of the residents were more related to the ability to decide how to live individually and socially than to the ability to control the physical environment.

### Psychosocial determinants of health

#### Social support

Social support was assessed through seven studies using a mixed methods design [12, 35, 45–49], including two with a comparison group [14, 50], nine qualitative methods [15, 16, 38–41, 51–53] and four quantitative methods [13, 37, 43, 54]. Two studies with a comparison group [14, 37] and three studies that compared the actual situation with previous situation [43, 46, 48] reported that social support was more evident in the cohousing model. In addition, the residents’ social networks were strengthened after they moved into cohousing [45].

Most studies reported that the cohousing model had beneficial effects on the residents’ social support. This effect could be found both in senior [12, 13, 35, 39, 41, 45, 47–50, 52, 54] and intergenerational projects [14–16, 37, 40, 43, 46, 53, 54]. One study found beneficial effects of social support among same-generation residents while intergenerational social support was less evident [51].

Three studies indicated less obvious effects. One studied a project targeting low-income women in which social support was not an immediate effect due to the diversity of the residents, although over time it seemed to be able to increase [38]. Other found no significant differences in the increase in social support comparing the perception of cohousing residents with the perception of residents in rental apartments with social activities. However, socialisation patterns were more open and autonomous among cohousing residents [52]. The third states that intergenerational solidarity was not evident [51].

Among studies reporting a beneficial effect on social support, three types of social support can be described: (a) instrumental (or functional) social support involving activities such as borrowing, housework, meal preparation, care during illness or childcare [12–14, 45, 47, 53]; (b) emotional support such as having close friendships, listening or providing support when someone had a personal problem [13, 35, 47]; and (c) recreational support, provided through different social activities organised by residents themselves [15, 50, 54].

#### Social isolation

The five studies assessing social isolation reported less loneliness among cohousing inhabitants using quantitative [13], mixed [48, 50] and qualitative methods [38, 52]. None incorporated a comparison group in the analysis. The four studies targeting the senior population reported that an active lifestyle in settings prevented social isolation and loneliness, which is a general problem among senior residents. Social interaction was enhanced by the architectural design of both indoor and outdoor common spaces, which was also described as an effective way to reduce social isolation [38, 48], in particular among elderly residents who were sick and frail [50]. However, it was not always experienced beneficially since privacy was valued as something that mattered [52].

#### Sense of community

Evidence was obtained through one study with quantitative methods [13], 6 using mixed methods [12, 14, 35, 47, 50, 55], including only one with a comparison group [14], and four qualitative designs [16, 38, 52, 53].

Several studies showed a beneficial influence of the cohousing model on the residents’ sense of community, both in senior [12, 13, 35, 47, 50, 52] and intergenerational projects [16, 53]. In contrast, one study about a project for low-income women showed detrimental effects on residents’ perception of sense of community. Moreover, another study reported contradictory effects. Living in cohousing increased the sense of community but could also be a source of struggle and fatigue to maintain it [14].

The studies reporting beneficial effects uncovered some sense of community-building pathways. For example, two studies reported that individuals intentionally chose the cohousing model in search of a sense of community [35, 50, 53]. In addition, they outlined the relevance of the residents’ engagement pathway throughout all stages of the cohousing development process as a critical source of community building, such as participation in the start-up stages in co-ownership projects [12, 16], self-management of common spaces and facilities [16] and the day-to-day community and mutual support [12, 47, 50, 55].

#### Sense of security

Nine studies examined the impact of cohousing on the sense of security [13, 16, 38, 45–49, 53]. All of them reported a positive association. The evidence was obtained from five studies using mixed methods [45–49], 3 qualitative studies [16, 38, 53] and one study using quantitative methods [13].

The sense of security gained was found among both senior [13, 45, 47, 48] and intergenerational projects [16, 38, 40, 46, 53]. The studies found that cohousing increased residents’ sense of security through both the physical and the social environment. In addition, it reduced residents’ sense of economic insecurity [38]. The physical aspects emphasised were open and well-lit spaces [16], safe children’s play areas [53] and a neighbourhood with a rich and pleasant atmosphere [38]. The social features that led to feelings of security were social relationships and trust [47], community coping [48] and social support among neighbours [13, 47, 48]. An economic sense of security among low-income women residents reduced stress, helplessness and frustration [38].

## Discussion

### Main findings

The purpose of this study was to examine all the known evidence on the relationship between communal living arrangements characterised as cohousing and health and wellbeing. Our review indicates that the cohousing model can be positively associated with health outcomes through psychosocial determinants of health, such as increased social support, sense of community and physical, emotional and economic security, as well as reduced social isolation. This association was more evident in cohousing models targeting the older population. Likewise, we found a limited number of studies assessing the direct health effects of the cohousing model. Some studies suggest that cohousing is positively associated with self-perceived physical and mental health outcomes and quality of life and wellbeing. However, extreme caution should be exercised in drawing any conclusions due to the dearth of data identified and the study designs used—mostly cross-sectional, with small samples or no comparison group—that precluded causal-based interpretations.

### What health outcomes have been studied in relation to cohousing?

With respect to the effect of cohousing on health outcomes, only 10 studies analysed the effect of cohousing on self-perceived health [35–38, 49] and quality of life and wellbeing [39–44].

The studies evaluating both health and quality of life showed relatively limited reproducibility and comparability. No other subjective health measures are available such as joy, happiness, sense of self-worth and value to others, or other measures related to stress and mental health. There is also a lack of evidence on other objective health measures such as the ability to perform physical, mental and social tasks or healthy behaviours. Other health measures that could be important to assess are health-related quality of life indices. For example, the EuroQoL-5 index is one of the most widely used instruments underpinning economic evaluations, which would allow quantification of quality of life and analysis of health effects in terms of the associated costs of this model compared with conventional housing arrangements.

### How could cohousing affect health, quality of life and wellbeing?

The cohousing model may be positively associated with health status through psychosocial pathways underlying health and illness. These mechanisms would be coherent with the evidence found in measures such as social support [12–16, 35, 37, 38, 40, 41, 43, 45, 47–54], sense of community [12, 16, 35, 38, 46, 47] and sense of security [13, 16, 38, 47, 48, 53]. This finding is consistent with other studies reporting the relationship between social support and health and wellbeing. For example, lack of a social network and support, social isolation and loneliness are linked to poor cardiovascular and mental health outcomes [56]. In contrast, living in a community characterised by higher levels of communication and mobilisation is positively associated with residents’ self-rated health status [57], especially in elderly persons. In addition, it has been shown that high social support and participation in social networks alleviates stress in older people, preventing them from developing functional decline [58] and mental health problems [59]. A sense of community has also been positively related to a range of health outcomes and indicators of wellbeing, including life satisfaction and loneliness [60], happiness [61], and quality of life [62].

There is a lack of studies aiming to identify the differential health and wellbeing effects resulting from cohousing models based on co-ownership. We found only one study that comparatively assessed the effect of tenure on quality of life [44]. That study observed no additional benefits among residents living in a model based on co-ownership tenure. We found some evidence to suggest that the co-ownership regime helped to increase autonomy and a sense of control among residents, which could enhance quality of life [42]. Further research is required on the potential health and quality of life gains among the different tenure arrangements in the cohousing model.

### Issues arising from the review

Over the last few years, cohousing has reappeared in various high-income countries. This has not gone unnoticed by several social sectors such as urbanists, politicians, social movements and non-profit organisations, with all of them showing a willingness to promote it. However, we cannot dismiss the possibility that the promotion of this model may increase social and health inequalities. Several studies discussed here [16, 52] have observed unequal access to cohousing projects. Populations from disadvantaged social classes would appear to have fewer opportunities to access them and thus less chance to benefit from the potential positive social and health effects. Therefore, we should not rule out the so-called paradox of promotion in public health, in which health promotion can have undesirable effects and increase health inequalities. This effect has been previously documented in a review of unintended harm in public health interventions [63]. Therefore, the unintended effects of cohousing on social and health inequalities should be considered by entities interested in promoting this model and in future research.

In this review, we found no evidence linking housing affordability and health among the cohousing experiences studied. However, there is some evidence that cohousing provides residential security as residents value living in a home at an affordable price for a long period of time [16, 38, 52]. Housing affordability is recognised as a material pathway to health, and there is substantial evidence linking housing affordability problems with a range of adverse health effects [3, 4] and health inequalities [64]. Potentially, cohousing has been considered a housing model that could help to decrease the commoditisation of housing, since it conceives housing as a social good that prioritises its use value over its exchange value. It is known that commodification of other key areas of a person’s life, such as food [65], care [66] or the health care system [67], among others, can lead to worse health. In general, in an intentional housing community, housing construction costs are often less subject to the capital gains of promoters; there is greater long-term stability of housing prices and mutual economic support that results in stable economic and social security for residents, who are less exposed to the precarious conditions of the neo-liberal housing market [18]. In this regard, there is a literature gap in relationship between cohousing and its potential effect on health at the individual and community level through socioeconomic aspects. Therefore, further research is required on the potential health gains associated with the affordability, stability, or collectivisation of economic uncertainties of cohousing living arrangements.

### Limitations and strengths

There is still no consensus on the definition of cohousing models. Some authors have attempted to define it and standardise its use for international communication avoiding the use of inconsistent and vague concepts [9]. This makes the search for evidence less complex and the comparative analysis more reliable. However, most of the studies selected were published before this effort at conceptualisation. Therefore, there may be a bias in the article selection due to divergence in search terms. However, the scoping review method used in this review is more flexible than a systemic review. This enabled us to have more flexible exclusion and inclusion protocols and to include articles that would have been ruled out using other review methods. Another limitation could be bias due to the use of language restrictions. Although the present review used the English, Spanish, French, German and Italian languages, cohousing has a long history in Denmark, and Danish was not a language covered in this review. However, to overcome this limitation, we contacted experts to find relevant references. The references gained did not allow us to identify new documents studying the relationship between cohousing and health, although additional information on the cohousing model was obtained that facilitated the discussion of the results obtained.

Despite these limitations, this review provides an important contribution to public health and social policies because, to the best of our knowledge, it is the first review to gather and systematise the scientific evidence related to this housing model, aiming to assess its health and welfare effects. The review also identifies knowledge gaps and could be used to inform future research. Likewise, gathering the present evidence will facilitate the design of evidence-based policies in the cohousing domain. In addition, a strong effort has been made to search for evidence by not limiting it to articles indexed in biomedical databases. The search was also performed in social science databases, enabling us to find articles that, although they were not focused on health effects, included them among their findings.

## Conclusions

This study examined the available evidence on cohousing from a public health perspective. The rationale is that housing is an important determinant of health and health inequalities, and cohousing is a potentially health-enhancing form of community living that raises many expectations for creating vivid social networks, communities and healthy environments. Various studies have provided a relatively consistent picture of the increased psychosocial health benefits of the community dimension and the emotional and social bond of this model of housing. However, more research is needed to address the knowledge gaps identified in this review. Future studies should measure health with objective and/or subjective health outcomes because most studies conducted to date have been performed in relation to psychosocial determinants of health. Furthermore, there is a need for studies with methodological approaches that provide clearer evidence of the effects of cohousing on health. Housing is a collection of components that together affect individuals’ lives. In that sense, other cohousing dimensions related to economic aspects, such as cost and stability, or environmental sustainability, their interactions and their impact on health and wellbeing, need to be explored in the future.

## Supplementary information

**Additional file 1.** Supplementary 1: Subject headings, keywords and search syntaxes. Supplementary 2: List of experts and organizations contacted

## Data Availability

Not applicable.
