# Supplementary material for: The effects of cohousing model on people’s health and wellbeing: a scoping review
Source: Public Health Rev. 2020 Oct 6;41:22. doi: 10.1186/s40985-020-00138-1 (PMC7539375; doi:10.1186/s40985-020-00138-1)
Supplement: Supplementary file 1 — Additional file 1. Supplementary 1: Subject headings, keywords and search syntaxes. Supplementary 2: List of experts and organizations contacted [file 40985_2020_138_MOESM1_ESM.docx]

**Supplementary Material Nº 1.** Subject headings, keywords and search syntaxes.

**Terms**

Cohousing:

Cohousing (MeSH), Collaborative housing, Collective housing, Communal housing, Housing co-op, co-operative housing, Intentional communities

Health outcomes and psychosocial determinants of health:

Health, Health status [MeSH], Perceived health, Self-reported health, Well-being, Quality of life, Mental health [MeSH], Psychiatric symptoms, Mental disorders [MeSH], Stress, Stress disorders [MeSH], Anxiety disorder [MeSH], Mood disorders [MeSH], Affective Symptoms [MesH], Depression [MeSH], Depress*, Hypertension [MeSH], Hypertens*, High blood pressure [MeSH], Blood pressure, Cardiovascular diseases [MeSH], Cardiovascular.

Psychosocial determinants of health:

Happiness, Positive Health, Life satisfaction, Social support, Social isolation, Sense of community.

**Syntaxes**

PubMed

(“cohousing” OR "collaborative housing" OR "collective housing" OR "communal housing" OR "housing co-op" OR “co-operative housing” OR “cooperative housing” OR "intentional communities") AND (“health” OR health status[MeSH Terms] OR “well-being” OR “wellbeing” OR “quality of life” OR mental health[MeSH Terms] OR mental disorders[MeSH Terms] OR "psychiatric symptom*" OR acute stress disorders[MeSH Terms] OR acute post-traumatic stress disorder[MeSH Terms] OR “stress” OR anxiety[MeSH Terms] OR anxiety disorders[MeSH Terms] OR “anxiety” OR mood disorders[MeSH Terms] OR "mood disorder*" OR affective disorders[MeSH Terms] OR "affective disorder*" OR affective symptoms[MeSH Terms] OR "affective symptom*" OR depression[MeSH Terms] OR depress* OR hypertension[MeSH Terms] OR hypertens* OR high blood pressure[MeSH Terms] OR "blood pressure*" OR cardiovascular diseases[MeSH Terms] OR cardiovascular OR “hapiness” OR “positive health” OR “life satisfaction” OR “social support” OR “social isolation” OR “sense of community”)

ProQuest databases (International Bibliography of the Social Science (IBSS), EconLit y ProQuest Health & Medical Complete)

(SU.EXACT("cohousing") OR SU.EXACT("collaborative housing") OR SU.EXACT("collaborative housing") OR SU.EXACT("communal housing") OR SU.EXACT (“housing co-op”) OR SU.EXACT (“co-operative housing”) OR SU.EXACT (“cooperative housing”) OR SU.EXACT (“intentional communities”)) AND (SU.EXACT("Health") OR SU.EXACT("Well-being") OR SU.EXACT("quality of life") OR SU.EXACT("Diseases") OR SU.EXACT("Mental health") OR SU.EXACT("Medical disorders") OR SU.EXACT("Mental depression") OR SU.EXACT("Anxieties") OR SU.EXACT("Anxiety") SU.EXACT("Mental illness") OR SU.EXACT("Mood disorders") OR SU.EXACT(“happiness”) OR SU.EXACT(“positive health”) OR SU.EXACT(“life satisfaction”) OR SU.EXACT(“social support”) OR SU.EXACT(“social isolation”) OR SU.EXACT(“sense of community”)

Scopus

(TITLE-ABS-KEY(cohousing OR "collaborative housing" OR "collective housing" OR "communal housing" OR "housing co-op" OR ” co-operative housing” OR ” cooperative housing” OR "intentional communities") AND (health OR well-being OR wellbeing OR “quality of life” OR "psychiatric symptom*" OR anxiety OR stress OR "mood disorder*" OR "affective disorder*" OR "affective symptom*" OR depress* OR hypertens* OR "blood pressure*" OR cardiovascular OR happiness OR “positive health” OR “social support” OR “social isolation OR “sense of community”))

Web of Science databases (Science Citation Index Expanded (ISI), Social Sciences Citation Index (SSCI) and SciELO)

(TS=cohousing OR TS="collaborative housing" OR TS="collective housing" OR TS="communal housing" OR TS="housing co-op" OR TS= co-operative housing OR TS= cooperative housing OR TS="intentional communities") AND (TS=health OR TS=well-being OR TS=quality of life OR TS=wellbeing OR TS="psychiatric symptom*" OR TS=anxiety OR TS=stress OR TS="mood disorder*" OR TS="affective disorder*" OR TS="affective symptom*" OR TS=depress* OR TS=hypertens* OR TS="blood pressure*" OR TS=cardiovascular OR TS=”happiness” OR TS=”positive health” OR TS=”social support” OR TS=”social isolation" OR TS= “sense of community”)

ScienceDirect

tak("cohousing" OR "collaborative housing" OR "collective housing" OR "communal housing" OR "housing co-op" OR "cooperative housing" OR "intentional communities")

JSTOR

("cohousing" OR "collaborative housing" OR "collective housing" OR "communal housing" OR "housing co-op" OR "cooperative housing" OR "intentional communities")

**Supplementary Material Nº 2. List of experts and organizations contacted**

**Experts**

Anne Labit - France

Angela Sanguinetti - USA

Neil Planchon - USA

Kerstin Kärnekull - Sweden

Henrik Gutzon Larsen - Denmark

Tobias Polsfuß - Germany

Jessica Lilli Köpcke - Germany

Raúl Aguayo-Krauthausen- Germany

**Organitzations**

Cohousing Research Network – USA and International

Habicoop -France

Wohnsinn.org - Germany

id22 - Germany

Cohousing-solutions. USA
